# π-Shape ESD Protection Design for Multi-Gbps High-Speed Circuits in CMOS Technology

**DOI:** 10.3390/ma16072562

**Published:** 2023-03-23

**Authors:** Chun-Rong Chang, Zih-Jyun Dai, Chun-Yu Lin

**Affiliations:** Department of Electrical Engineering, National Taiwan Normal University, Taipei City 106, Taiwan

**Keywords:** electrostatic discharge (ESD), silicon-controlled rectifier, π-shape circuit

## Abstract

CMOS integrated circuits are vulnerable to electrostatic discharge (ESD); therefore, ESD protection circuits are needed. On-chip ESD protection is important for both component-level and system-level ESD protection. In this work, on-chip ESD protection circuits for multi-Gbps high-speed applications are studied. π-shaped ESD protection circuit structures realized by staked diodes with an embedded silicon-controlled rectifier (SCR) and resistor-triggered SCR are proposed. These test circuits are fabricated in CMOS technology, and the proposed designs have been proven to have better ESD robustness and performance in high-speed applications.

## 1. Introduction

The required operating speed of integrated circuits is increasing, and multi-Gbps high-speed CMOS ICs are widely used in digital communication systems [1]. However, the transistors are weak to electrostatic discharge (ESD) events in CMOS technology [2,3]. In order to pass the component-level ESD test [4], all integrated circuits should be designed with an on-chip ESD protection circuit. To achieve whole-chip ESD protection, the ESD protection at I/O and the power-rail ESD clamp circuit should be put between VDD and VSS to provide the ESD current paths. The on-chip ESD protection scheme can provide sufficient ESD robustness for the component-level test, but the system-level ESD should also be considered [5]. In order to suppress the system-level ESD, the off-chip ESD protection design will be used but some energy cannot be released. The on-chip ESD protection will also help with system-level ESD protection. Therefore, on-chip ESD protection is important for both component-level and system-level ESD protection. In high-speed applications, the ESD protection device may affect circuit performance via its parasitic capacitance at the I/O pad [6,7]. Thus, reducing the parasitic capacitance is one of the most important considerations for high-speed applications [8,9].

**Figure 1:**
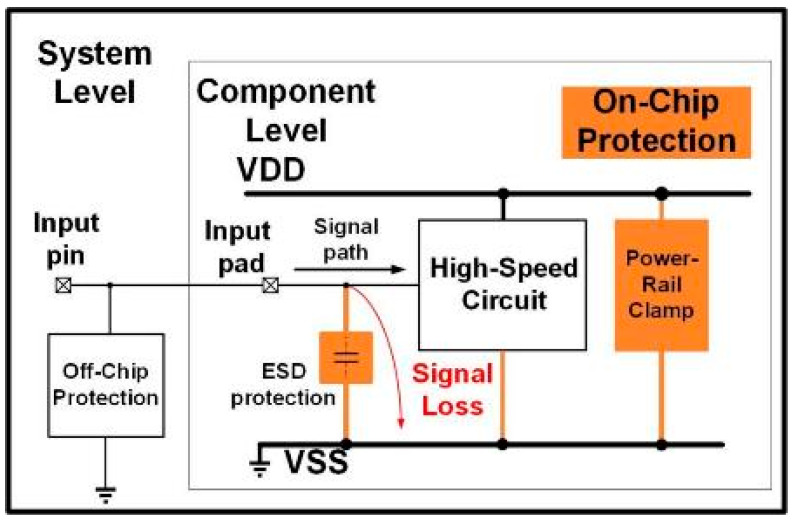
Whole-chip ESD protection block and signal loss caused by ESD protection device at input pad in the high-speed application.

The whole-chip ESD protection block and signal loss caused by the ESD protection device from input pad to ground are shown in Figure 1, and the signal integrity will be degraded. In order to provide a good ESD robustness for high-speed circuits and reduce the effect of the parasitic capacitance of ESD protection devices, the diode and silicon-controlled rectifier (SCR) are usually used [10,11]. However, the parasitic capacitance of a diode or SCR may still be too large to be tolerable for higher-frequency applications. Therefore, to cancel the parasitic capacitance of ESD applications, the inductor is usually added to the ESD protection circuit. For example, the T-coil-based ESD protection circuit for 5 GHz LNAs has been presented [7]. This design has achieved 2 kV human body model (HBM) ESD robustness with 2 dB degradation. However, the T coil will occupy a large area. Another method is to reduce the parasitic capacitance of devices. Some SCRs have been designed for ESD protection with about 100 fF, 59 fF, and 25 fF parasitic capacitance [9,12,13]. However, these designs have not been applied to protect high-frequency circuits. For broadband applications, π-diode can suit I/O protection with bandwidth within 0~20 GHz [14]. Another approach for broadband application is π-MOS. With the proper matching with an inductor, the π-MOS has been applied to protect the 6.7~15.3 GHz LNAs [15]. All these designs can achieve ESD protection requirements, but these designs still have quite parasitic capacitance. The capacitance will cause high-frequency signal loss and degrade the performance of the core circuit in high-frequency applications. In this work, the proposed π-stacked diode with embedded SCR (π-SDSCR) and π-resistor triggered SCR (π-RTSCR) and the traditional π-shape ESD protection circuits are studied in CMOS technology to compare their signal loss and ESD robustness.

## 2. Design of π-Shape ESD Protection Circuit

### 2.1. π-Shape Circuit

In on-chip ESD protection design, the ESD protection circuit should provide the ICs with a strong ability to suppress ESD stress. Thus, the ESD protection circuit must be large to endure the high-current situation. However, a larger size for protection devices will result in higher parasitic capacitance and lead to extra signal loss. To overcome this issue, ESD protection circuits for high-speed applications are splitting ESD protection circuits into multiple sections [16,17]. This method can hold the ESD robustness and cause lower high-frequency degradation. To accomplish better high-frequency performance, all the protection devices in each section are connected with an inductor. Via proper impedance matching, the inductor will resonate with the parasitic capacitance of ESD protection devices. Therefore, the transmitted signal has the lowest high-frequency loss and provides sufficient ESD robustness.

**Figure 2:**
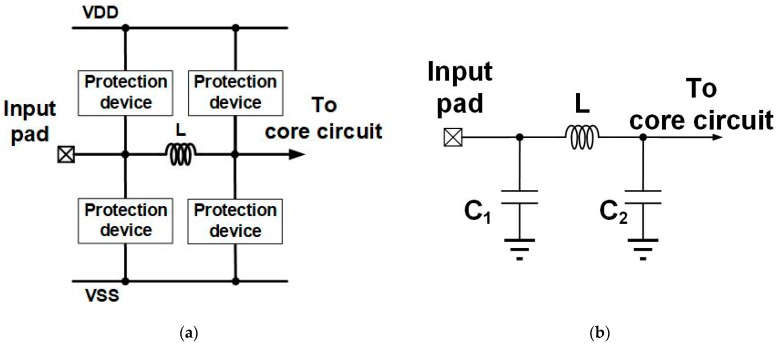
(**a**) π-shape ESD protection circuit and (**b**) equivalent circuit of π-shape ESD protection circuit.

Figure 2a,b show the two-section ESD protection circuit and its equivalent circuit. C_1_ and C_2_ are the parasitic capacitance of ESD protection devices in two stages, such as diode or SCR. The parasitic capacitance of the ESD protection device from the input pad to VSS and input pad to VDD are parallel in small-signal model analysis. The size of the protection device will determine the value of parasitic capacitance.

### 2.2. Traditional π-Diode and π-MOS

**Figure 3:**
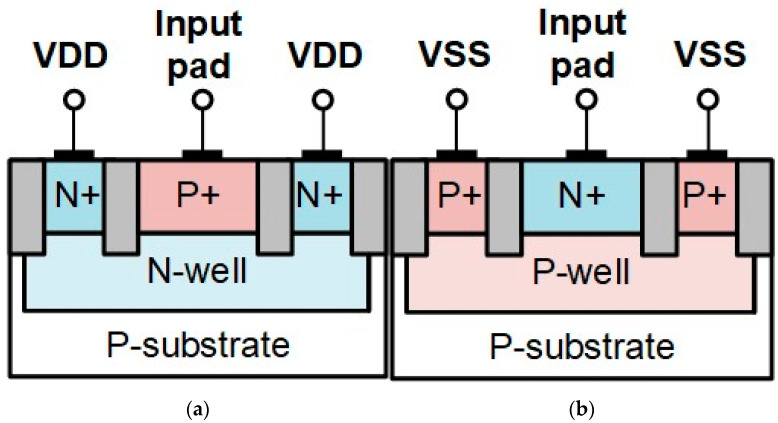
Cross-sectional view of symmetrical diode: (**a**) P-type and (**b**) N-type.

In CMOS technology, the diode is a general solution for ESD protection. Two types of diodes are usually used. As shown in Figure 3a, the anode of the P-type diode is the P+, and the cathode is the N-well. The anode of the N-type diode is the P-well, and the cathode is N+, as shown in Figure 3b. In high-speed applications, the ESD diode can suit the parasitic capacitance requirement and provide enough ESD robustness [18]. The diode will turn on under forward bias; then, it can discharge the ESD current at a low holding voltage.

**Figure 4:**
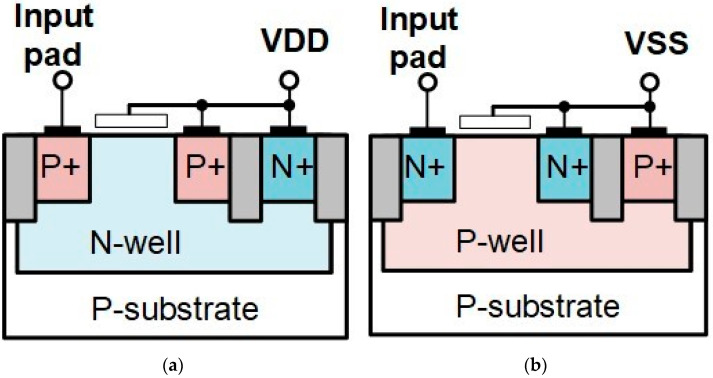
Cross-sectional view of MOSFET: (**a**) GDPMOS and (**b**) GGNMOS.

The MOSFET device is another ESD protection device in CMOS technology [19]. When the MOSFET is connected as gate-grounded NMOS (GGNMOS) or gate-VDD PMOS (GDPMOS), it becomes a two-terminal device and forms parasitic capacitance between two terminals as anode and cathode. This device can discharge ESD current through its parasitic n-p-n or p-n-p bipolar transistor. The cross-section view of GGNMOS and GDPMOS is shown in Figure 4a,b. In order to match the ESD design rules, the gate length of MOSFET is usually larger than two-times the smallest gate length in process limitation. The clearance from poly to contact on the source and drain sides is designed according to the ESD rules, and the silicide blocking layer is also used on the drain side of MOSFET to increase the ESD robustness.

### 2.3. Proposed π-SDSCR and π-RTSCR

**Figure 5:**
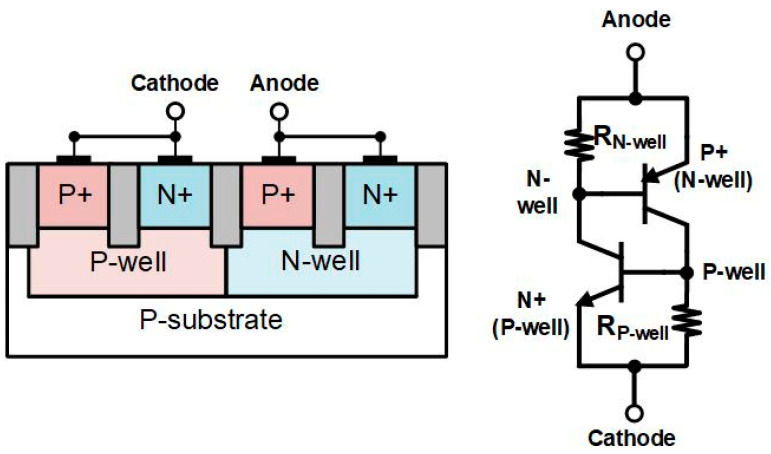
Cross-sectional view and schematic of SCR.

The silicon-controlled rectifier (SCR) is a semiconductor device formed by p-n-p-n (P+/N-well/P-well/N+) in CMOS technology. The cross-section view and schematic of SCR are shown in Figure 5. In high-speed applications, the parasitic effect is a very important issue, and several low-capacitance devices have been reported [9,12]. However, these devices are not applied in the high-speed application. In this work, the SDSCR and RTSCR combine with the inductor of TIA to form a distributed ESD protection circuit. It does not need any extra components to form a protection circuit.

**Figure 6:**
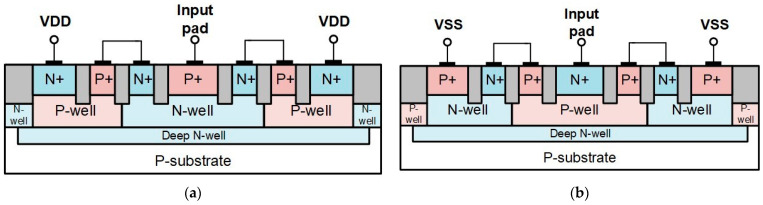
Cross-sectional view of SDSCR: (**a**) P-type and (**b**) N-type.

As shown in Figure 6a,b, the cross-section view of p-type and n-type stacked diodes with embedded SCR (SDSCR) is two stacked diodes. The terminal connected to the input pad should be placed in the center of the device structure to reduce the parasitic capacitance. In Figure 6a, the P+ of p-type SDSCR is connected to the input pad and the N+ is connected to VDD. In Figure 6b, the N+ of n-type SDSCR is connected to the input pad and the P+ is connected to VSS. When ESD occurs, the ESD current will flow into the input pad. These diodes will be turned on to discharge current first; then, the embedded SCR will finish the rest of the ESD current.

**Figure 7:**
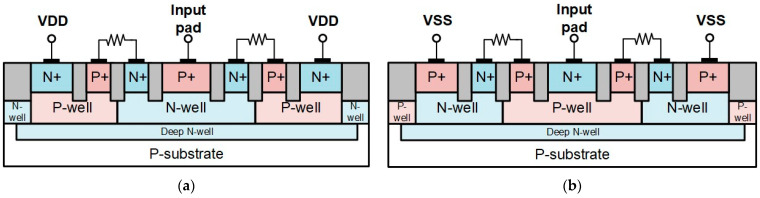
Cross-sectional view of RTSCR: (**a**) P-type and (**b**) N-type.

Different from SDSCR, the resistor-triggered SCR (RTSCR) is triggered by a small resistor of about 100~200 Ω. The resistor of RTSCR is used to prevent the stacked diode that might be damaged by the ESD current and force the current flowing into the SCR path. The value of the resistor in this process is not very precise. Thus, the value of the resistor in the paragraph is described as an interval of about 100~200 Ω. Cross-sectional views of p-type and n-type RTSCR are shown in Figure 7a,b, respectively. It has been reported as a low-capacitance ESD protection device [20]. Like SDSCR, the terminal connected to the input pad should also be placed in the center of the structure. When the ESD occurs, the current will flow into VDD or VSS from the input pad. Due to the small resistor, the current can be drawn from the N-well to P-well. This mechanism can help the SCR turn on faster to discharge current.

## 3. Implementation and Measurement Results

### 3.1. Test Device Implementation

**Figure 8:**
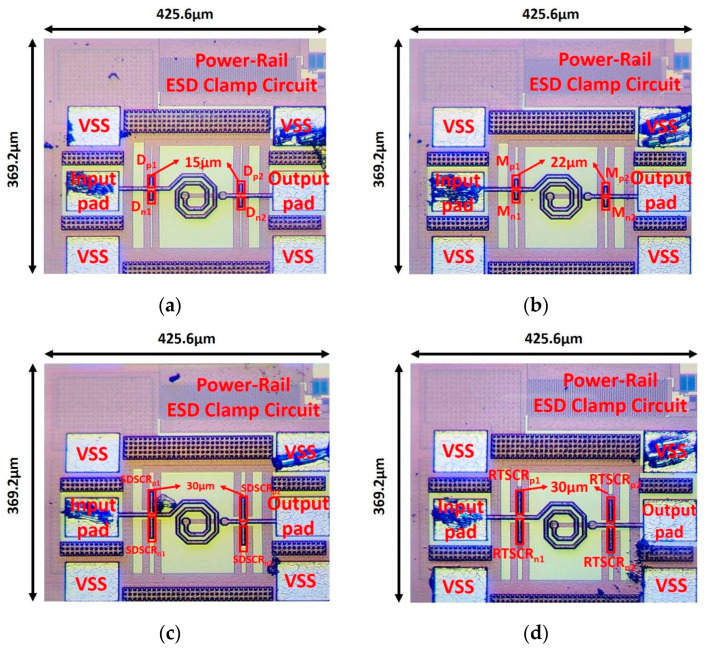
Chip photo of π-shape ESD protection circuits: (**a**) traditional π-diode, (**b**) traditional π-MOS, (**c**) proposed π-SDSCR, and (**d**) proposed π-RTSCR.

In this work, 0.18 μm CMOS technology was used to implement the test key of π-shape circuit with the traditional device and proposed device. All the ESD devices are designed to have the same total parasitic capacitance. The sizes of the diode, MOS, SDSCR, and RTSCR are selected to be 30 μm, 44 μm, 60 μm, and 60 μm, respectively. Then, these ESD devices are divided into two stages and matched with an inductor between two stages. The parasitic capacitance will resonate with the inductor to achieve the lowest signal loss. In order to endure the ESD current, the line width of the inductor should be wider. The metal width of the inductor is chosen as 6 μm. The chip photos of test devices are shown in Figure 8a–d. These devices contain a matching inductor between stage 1 and stage 2 of the protection device. The power clamp circuit is used in these protection designs to accomplish whole-chip ESD protection. The p-type devices (D_P1_, D_P2_, M_P1_, M_P2_, SDSCR_P1_, S-DSCR_P2_, RTSCR_P1_, and RTSCR_P2_) are connected to VDD from the input pad by using metal 3 and metal 4, then providing the path for ESD current. The n-type devices (D_N1_, D_N2_, M_N1_, M_N2_, SDSCR_N1_, SDSCR_N2_, RTSCR_N1_, and RTSCR_N2_) are connected to VSS from the input pad by using metal 2 and metal 1. The parameters of each device and element are listed in Table 1. The inductor is implemented by using metal 6 (top metal) and the inductance is chosen as 0.31 nH.

### 3.2. High-Frequency Performance

**Figure 9:**
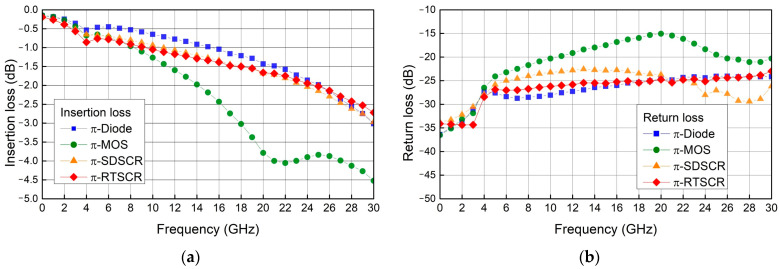
Measured insertion loss and return loss of π-shape ESD protection circuits: (**a**) insertion loss (**b**) return loss.

To confirm the high-frequency performance of π-shape ESD protection circuits, the S parameters are measured by using an on-wafer measurement system. Figure 9a,b show the measured S_21_ (insertion loss) and S_11_ (return loss) of each π-shape circuit. The proposed π-SDSCR and π-RTSCR are slightly different from the π-diode, and the π-MOS circuit is worse than the others. The S_21_ of π-diode, π-MOS, π-SDSCR, and π-RTSCR at 20 GHz is −1.43 dB, −3.79 dB, −1.68 dB, and −1.66 dB, respectively. The S_11_ of π-diode, π-MOS, π-SDSCR, and π-RTSCR at 20 GHz is −24.6 dB, −15.0 dB, −23.7 dB, and −24.7 dB, respectively. From the measurement results, the insertion loss of π-SDSCR and π-RTSCR is lower than 2 dB, which means the π-SDSCR and π-RTSCR are better choices than π-diodes and π-MOS for high-speed applications.

### 3.3. Human Body Model (HBM) ESD Test

To obtain the ESD robustness of π-shape ESD protection circuits, the HBM test should be executed. In order to confirm the failure of ESD protection devices, the criterion is defined as the leakage current increasing about 30%. The HBM test results are listed in Table 2. The HBM measurement results of π-diode, π-MOS, π-SDSCR, and π-RTSCR are 4 kV, <1 kV, >8 kV, and >8 kV, respectively.

### 3.4. Human-Metal-Model (HMM) ESD Test

The human-metal mode (HMM) test method to investigate the robustness under ESD gun stress is investigated [21,22]. The HMM measurement results of each mode are listed in Table 3. The HMM measurement results of π-diode, π-MOS, π-SDSCR, and π-RTSCR are 1.6 kV, 0.3 kV, 2.9 kV, and 2.7 kV, respectively.

### 3.5. Transmission-Line-Pulsing (TLP) Measurement

**Figure 10:**
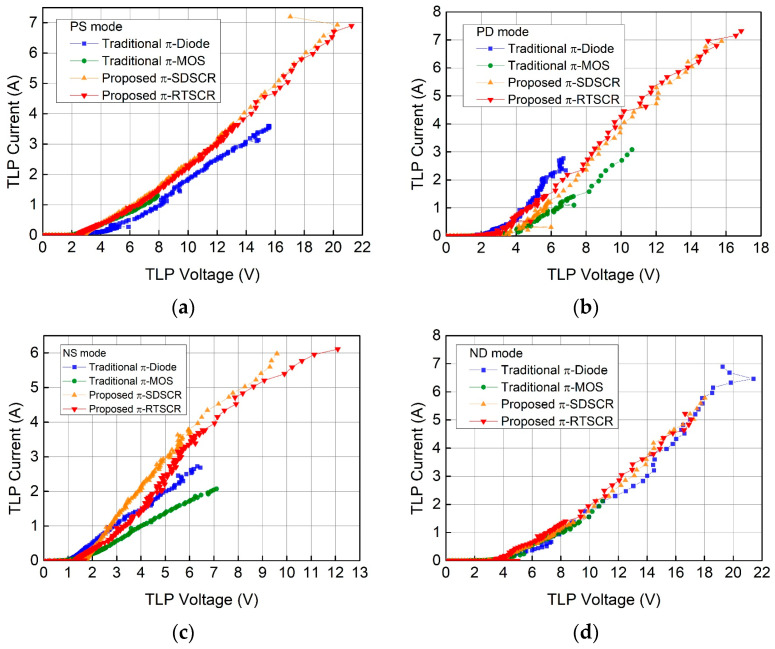
TLP-measured I–V characteristics of π-shape ESD protection circuits: (**a**) PS mode (**b**) PD mode (**c**) NS mode (**d**) ND mode.

To capture the I–V characteristic of these ESD protection devices, transmission-line-pulsing (TLP) system is used to get these parameters. Each ESD protection device should be measured in PS mode, PD mode, NS mode, and ND mode. Figure 10a–d are the TLP measurement results in each mode. The second breakdown current (I_t2_) is listed in Table 4. The TLP measurement results π-diode, π-MOS, π-SDSCR, and π-RTSCR in the worst case are 2.68 A, 1.29 A, 5.78 A, and 5.02 A, respectively. From the measurement results, the proposed designs are better than traditional designs.

### 3.6. Figure of Merit (FOM)

To verify the difference between traditional and proposed design, the figure of merit (FOM) [23] is defined as

(1) $${FOM}_{\pi \text{-}device}=\frac{I_{t2}}{\left|Insertion\;Loss\right|*parasitic\;capacitance\;per\;width}$$

where I_t2_ is the lowest secondary breakdown current in the TLP test, insertion loss is measured insertion loss at 20 GHz. The operation frequency is selected at 20 GHz because the ESD protection circuit is used for 40 Gb/s high-speed application. Because the HBM level of SDSCR and RTSCR are higher than 8 kV, the index of FOM_π-device_ is selected I_t2_ instead of HBM level and the input capacitance is taken account into FOM_π-device_. The comparison results are listed in Table 5. Considering the FOM_π-device_ of π-diode, π-MOS, π-SDSCR, and π-RTSCR are 1.71, 0.30, 2.45, and 1.82, respectively. Obviously, the proposed π-SDSCR and π-RTSCR are better than the traditional designs.

## 4. Application of π-Shape ESD Protection Circuits

### 4.1. Trans-Impedance Amplifier (TIA)

**Figure 11:**
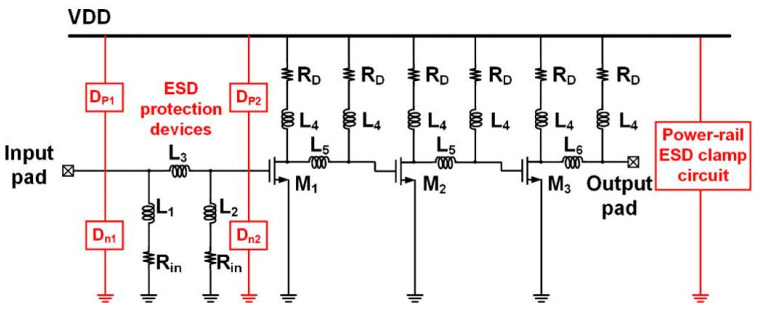
The architecture of TIA with π-shape ESD protection circuit.

In high-speed applications, the transmission speed is increasing rapidly. The data rate of optical communication system is also pushed up. To keep the signal integrity, the core circuit should have large bandwidth. Trans-impedance amplifier is an important role in the high-speed communication system. It can provide a large enough bandwidth and transfer the current signal to voltage signal. Figure 11 shows the architecture of TIA with π-shape ESD protection circuit. The π-shape ESD protection circuits are equipped in the input and match with the input-matching inductor. The power clamp is put between power line (VDD) and the ground line (VSS) to achieve whole-chip ESD protection.

### 4.2. High-Frequency Performance

**Figure 12:**
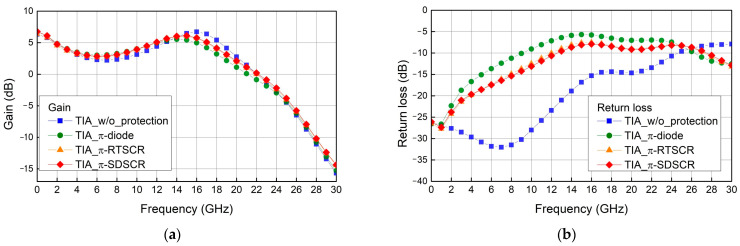
Measurement result of TIA with π-shape ESD protection: (**a**) gain and (**b**) return loss.

To verify the effect of π-shape ESD protection circuit, the TIA circuit is equipped with the π-diode, π-SDSCR, and π-RTSCR. The high-frequency performances are measured by 67 GHz RFIC parameter measurement system with 2-port GSG probes. The TIA circuit is composed of three stages common source amplifier. The supply voltage is 1.8 V, and the input bias is 1 V. The drain resistors are selected as 180 Ω, and the input resistors are chosen as 100 Ω. The inductors L_1_ and L_3_ = 0.46 nH, L_2_ = 0.76 nH, L_4_ = 1.6 nH, L_5_ = 1.2 nH, and L_6_ = 0.22 nH. As shown in Figure 12a,b, the high-frequency performance is not degraded too much by π-shape ESD protection circuits.

### 4.3. Transmission-Line-Pulsing (TLP) Measurement

**Figure 13:**
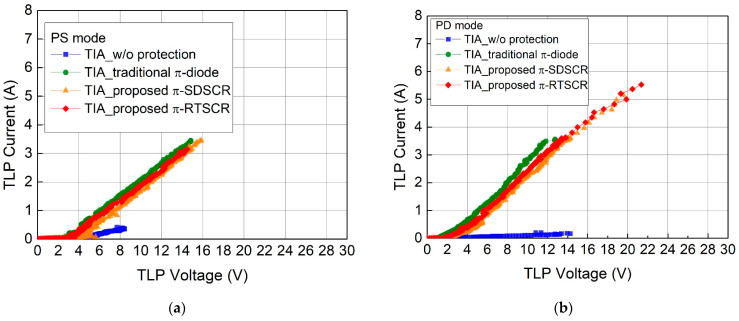

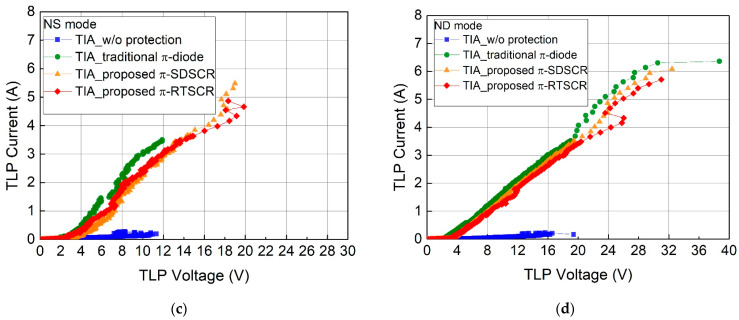
TLP-measured I–V characteristics of TIA with/without π-shape ESD protection circuits: (**a**) PS mode (**b**) PD mode (**c**) NS mode (**d**) ND mode.

Figure 13a–d are the TLP measurement result in each mode. The second breakdown current (I_t2_) is listed in Table 5. The TLP measurement results of TIA w/o protection, TIA with traditional π-diode, TIA with proposed π-SDSCR, and TIA with proposed π-RTSCR in the worst case are 0.40 A, 3.44 A, 3.45 A, and 3.14 A, respectively. The weakest discharging mode is PS mode.

### 4.4. Human Body Model (HBM) Measurement

The HBM test results are listed in Table 6. The HBM measurement results of TIA without protection, TIA with traditional π-diode, TIA with proposed π-SDSCR, and TIA with proposed π-RTSCR are 0.4 kV, 6 kV, 7 kV, and >7.5 kV, respectively. The weakest discharging mode is PS mode.

### 4.5. High-Frequency Performance after HBM Test

**Figure 14:**
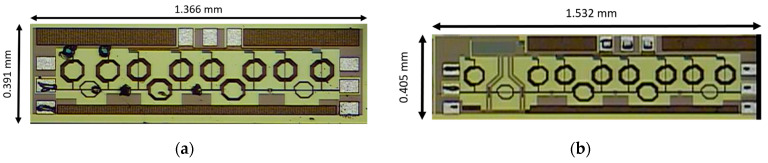
Photo of TIA circuit after HBM test: (**a**) TIA without protection; (**b**) TIA with proposed π-SDSCR.

**Figure 15:**
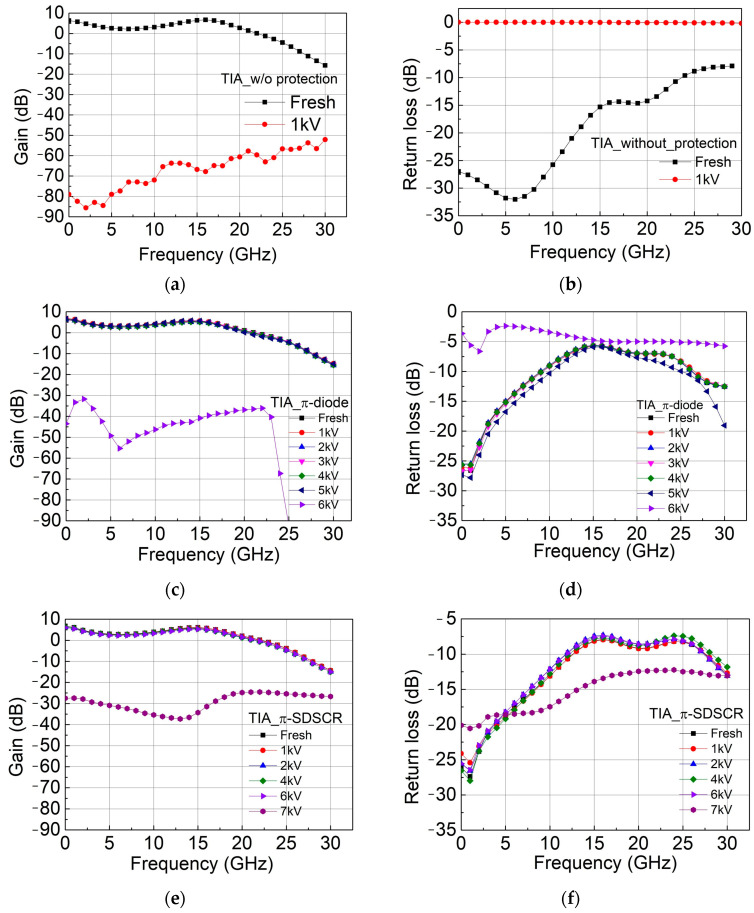

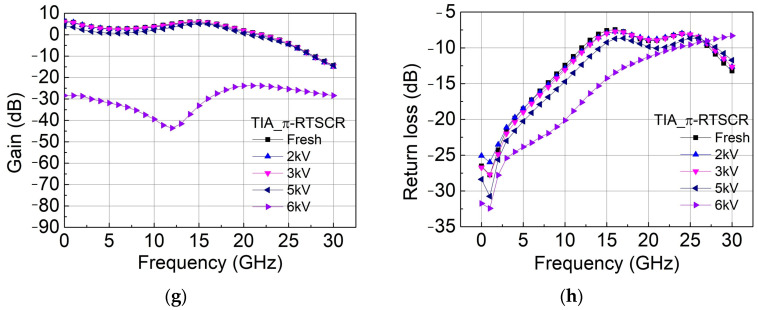
High-frequency performance of each TIA circuit with/without protection circuit after HBM test: (**a**) gain of TIA without protection (**b**) return loss of TIA without protection (**c**) gain of TIA_π-diode (**d**) return loss of TIA_π-diode (**e**) gain of TIA_π-SDSCR (**f**) return loss of TIA_π-SDSCR (**g**) gain of TIA_π-RTSCR (**h**) return loss of TIA_π-RTSCR.

The failure criterion of leakage current does not clearly show whether the circuit is damaged or not because the leakage current in the input stage is large. Another method is taking a photo under the microscope. As shown in Figure 14a, the TIA without protection is damaged after the 1 kV HBM test. However, in Figure 14b, the TIA with the proposed π-SDSCR ESD protection circuit was not obviously damaged under the microscope. In order to find the correct HBM level, the high-frequency performance after HBM test should be measured. The measurement results of each TIA circuit with/without protection circuit are shown in Figure 15a–h. The HBM level of TIAs after HBM test of TIA without protection, TIA with π-diode, TIA with π-SDSCR, and TIA with π-RTSCR are <1 kV, 5 kV, 6 kV, and 5 kV, respectively.

### 4.6. Figure of Merit (FOM)

To verify the performance of TIAs with/without protection circuit, the figure of merit (FOM_TIA_) [23] is defined as

(2) $${FOM}_{TIA}=\frac{Gain*HBM\;level}{parasitic\;capacitance\;per\;width}$$

where HBM level is the lowest HBM level in the HBM test. Gain is measured gain of TIA at 17 GHz, and the input capacitance is taken account in FOM_TIA_. The operation frequency is selected at 17 GHz because the function of TIAs are only available from 0 to 17 GHz. The comparison results are listed in Table 7. Considering the FOM_TIA_ of TIA without protection, TIA traditional π-diode, TIA with proposed π-SDSCR, and TIA with proposed π-RTSCR are 6.38, 21, 29.88, and 25.45, respectively. With this result, the proposed TIA with proposed π-SDSCR and TIA with proposed π-RTSCR are better than TIA with traditional π-diode and TIA without protection.

## 5. Conclusions

In this work, the π-shape ESD protection circuits are proposed for high-speed application in the CMOS process. The parasitic capacitance of the proposed design per width (2 fF/um) is lower than the traditional design. From the high-frequency measurement result, the insertion loss of each π-shape ESD protection circuit is higher than −2 dB, expect the π-MOS. In addition, the return loss is lower than −20 dB. It is suited to high-frequency/speed operations with less signal loss. In the TLP test, the I_t2_ of proposed π-SDSCR and proposed π-RTSCR is higher than the traditional π-diode and traditional π-MOS. The same results in the HBM and HMM results are shown. According to the FOM, the performance of the proposed designs is better for ESD protection. The performances of TIA with the proposed ESD protection design are also verified in this work.

## Figures and Tables

**Table 1:** Parameters of ESD devices and matching inductors.

Test Device	Parasitic Capacitance of ESD Device (fF)	ESD Device Width (μm)	Inductor (nH)
Traditional π-Diode	~120	30	0.31
Traditional π-MOS	~120	44	0.31
Proposed π-SDSCR	~120	60	0.31
Proposed π-RTSCR	~120	60	0.31

**Table 2:** HBM measurement results of π-shape ESD protection circuits.

Test Device	HBM (kV)			
	PS Mode	PD Mode	NS Mode	ND Mode
Traditional π-Diode	5	5	4	>8
Traditional π-MOS	<1	6	3	2
Proposed π-SDSCR	>8	>8	>8	>8
Proposed π-RTSCR	>8	>8	>8	>8

**Table 3:** HMM measurement results of π-shape ESD protection circuits.

Test Device	HMM (kV)			
	PS Mode	PD Mode	NS Mode	ND Mode
Traditional π-Diode	2.6	2.3	1.6	3.6
Traditional π-MOS	0.3	1.2	1.2	1
Proposed π-SDSCR	3	2.9	3.3	3.4
Proposed π-RTSCR	2.7	2.8	3.7	3.1

**Table 4:** FOM comparison.

Test Device	I_t2_ (A)	Loss (dB)	Input Capacitance (fF/μm)	FOM $\left(\frac{A\times \mu m}{fF\times dB}\right)$
Traditional π-Diode	2.68	−1.43	4	0.43
Traditional π-MOS	1.29	−3.79	2.72	0.11
Proposed π-SDSCR	5.78	−1.68	2	1.22
Proposed π-RTSCR	5.02	−1.66	2	0.91

**Table 5:** I_t2_ measurement results of TIA with/without π-shape ESD protection circuits.

Test Circuit	I_t2_ (A)			
	PS Mode	PD Mode	NS Mode	ND Mode
TIA w/o protection	0.40	0.17	0.23	0.16
TIA with traditional π-diode	3.44	3.55	3.50	6.36
TIA with proposed π-SDSCR	3.45	5.15	5.48	6.08
TIA with proposed π-RTSCR	3.14	5.52	4.86	5.71

**Table 6:** HBM measurement results of TIA with/without π-shape ESD protection circuits.

Test Circuit	HBM (kV)			
	PS Mode	PD Mode	NS Mode	ND Mode
TIA w/o protection	0.8	0.6	1	0.4
TIA with traditional π-diode	6.1	6	6	>8
TIA with proposed π-SDSCR	>8	7	>8	>8
TIA with proposed π-RTSCR	>8	7.5	>8	>8

**Table 7:** Comparison of each TIA circuit with different protection circuit.

Test Circuit	HBM (kV)	Gain (dB)@17 GHz	Input Capacitance (fF/μm)	FOM_TIA_ $\left(\frac{kV\times dB\times \mu m}{fF}\right)$
TIA without protection	<1	6.38	N/A	6.38
TIA with traditional π-diode	5	4.20	2.72	7.72
TIA with proposed π-SDSCR	6	4.98	2	14.94
TIA with proposed π-RTSCR	5	5.09	2	12.73

## Data Availability

Not applicable.
